# Long-Term Cardiovascular and Metabolic Health Outcomes of Gestational Diabetes Mellitus: A Systematic Review

**DOI:** 10.7759/cureus.79555

**Published:** 2025-02-24

**Authors:** Inayat Ullah, Samad Ali Khan, Dure Nayab, Sohail Ahmad, Asmad Khan, Nughman Ali, Jamil Ahmad, Zeeshan Ali, Sundas Safdar, Muhammad Imran Khan

**Affiliations:** 1 General Medicine, Lady Reading Hospital Peshawar, Peshawar, PAK; 2 General Surgery, Peshawar Institute of Medical Sciences, Peshawar, ESP; 3 Internal Medicine, Lady Reading Hospital Peshawar, Peshawar, PAK; 4 Internal Medicine, Hayatabad Medical Complex, Peshawar, PAK; 5 Nephrology Medicine, One Brooklyn Hospital, New York City, USA; 6 Medicine and Surgery, Liaquat College of Medicine and Dentistry, Karachi, PAK; 7 Diagnostic Radiology, Lady Reading Hospital Peshawar, Peshawar, PAK; 8 Dermatology, Combined Military Hospital Peshawar, Peshawar, PAK

**Keywords:** cardiovascular outcomes, gestational diabetes mellitus, long-term effects, metabolic health, systematic review

## Abstract

The public health concern associated with gestational diabetes mellitus (GDM) generates serious cardiovascular and metabolic impacts that affect both mothers whose condition went undiagnosed and their offspring. The review evaluates contemporary data about how GDM affects long-term health results. We searched published studies from databases such as PubMed, Scopus, and the Cochrane Library. A total of 710 records were identified, with 173 duplicates and 61 irrelevant records removed, leaving 476 for screening. After excluding 282, 194 reports were sought for retrieval, 97 were not retrieved, 97 were assessed for eligibility, 85 were excluded, and 12 studies were included in the review. Women with GDM face higher risks of type 2 diabetes (HR 2.5-7.1), hypertension (HR 3.2), and metabolic syndrome (HR 4.1). Their offspring have increased risks of obesity (OR 2.9), insulin resistance, cardiovascular disease, and metabolic disorders from childhood to adolescence. This review examines early detection biomarkers alongside strategies like lactation, probiotics, and CRP levels to potentially reduce maternal GDM risks. Although research had some methodological limitations regarding diagnostic irregularities and variable follow-up durations the findings demonstrate that early identification of GDM matters alongside customized care plans and long-term monitoring of women's health status. The review offers vital clinical guidelines and research pathways for advancing maternal and child health practices.

## Introduction and background

The medical condition known as gestational diabetes mellitus (GDM) reveals glucose intolerance when it is diagnosed for the first time during pregnancy. GDM affects 14% of all pregnancies worldwide and continues to grow as a vital public health issue [1-3]. The medical conditions resulting from GDM have important repercussions for both maternal health and fetal development. Women diagnosed with GDM have a sevenfold increased risk of developing type 2 diabetes, particularly in middle and late adulthood [4,5]. Their offspring face a sixfold higher risk of type 2 diabetes and are more prone to metabolic disorders from childhood through adulthood [6-8].

The pathophysiological process of GDM stems from insulin resistance and pancreas β-cell dysfunction. Pregnancy-related hormonal fluctuations create insulin resistance, but expectant mothers normally amplify their insulin output to preserve balance. Women affected by GDM experience a deterioration of their insulin compensatory capability which triggers hyperglycemia and additional metabolic abnormalities [8,9]. Researchers must focus on discovering biochemical identifiers that can forecast GDM development and related medical complications [10].

The effects of GDM extend well beyond harming pregnant mothers. Epidemiological studies demonstrate that fetuses who experience hyperglycemia develop impaired metabolic programming which results in lasting health complications [11]. These children show an increased susceptibility to obesity alongside hypertension and cardiovascular disease thus stressing the significance of preventive measures and early interventions [12,13].

Numerous research efforts into GDM remain inconsistent regarding diagnostic definitions while treatment plans and sustained care strategies show varying results. GDM diagnosis criteria differ across international guidelines because the American Diabetes Association (ADA) and the International Association of Diabetes and Pregnancy Study Groups (IADPSG) use different thresholds for glucose measurements [14]. Diagnostic criteria discrepancies create barriers for researchers who need to compare research results along with the development of standardized therapeutic practices.

The present systematic review investigates how GDM exposure affects metabolic and cardiovascular health both during and following the pregnancy period. This review combines evidence gathered from randomized controlled trials (RCTs) as well as cohort studies and observational studies to deliver an extensive overview of GDM's impacts on maternal and child health. The review examines both predictive biomarkers for unfavorable results and intervention strategies to minimize GDM-linked complications [15].

The study investigated lactation's impact on metabolic risks and its relationship to postpartum glycemic control management along with CRP's predictive value for future metabolic health outcomes [16,17]. The research produces subgroup analysis results to understand how risk levels differ according to population demographics, study approaches, and intervention methods. Meta-analytic approaches combine individual risk data into consolidated risk estimates, which enhance understanding of GDM's long-term health impact [18].

This systematic review adds value to existing knowledge through its emphasis on detecting GDM early and providing person-focused care and sustained follow-up for previously affected women. The findings underline a need for policy-based solutions that establish ongoing cardiovascular and metabolic assessments as a standard practice in post-GDM populations. Research findings will shape clinical practice decisions in addition to guiding future investigations within maternal and child health domains.

## Review

Methodology

The current study evaluates existing research data to analyze how GDM affects heart health and metabolic well-being in both pregnant mothers and their offspring over the long term. This review adopted established guidelines from systematic reviews which maintain transparency and provide reproducible results while enabling a thorough analysis of the subject matter.

Literature Search Strategy

Researchers conducted their literature search by utilizing predefined criteria across PubMed and Scopus together with Web of Science and Cochrane Library. A comprehensive database search was used for Boolean operators and MeSH terms such as “Gestational Diabetes Mellitus,” and “long-term outcomes” and “cardiovascular health” and “metabolic health” and “systematic review” search terms in various combinations. The review incorporated research published from January 2014 to December 2024. Studies that met the inclusion criteria consisted of papers exclusively written in English with human research subjects. The analysis included research exploring the long-term cardiovascular and metabolic health impacts of GDM on both maternal and offspring systems. Our review excluded research with inadequate information and studies from animals and primary data-void reviews.

Study Selection

**Figure 1:**
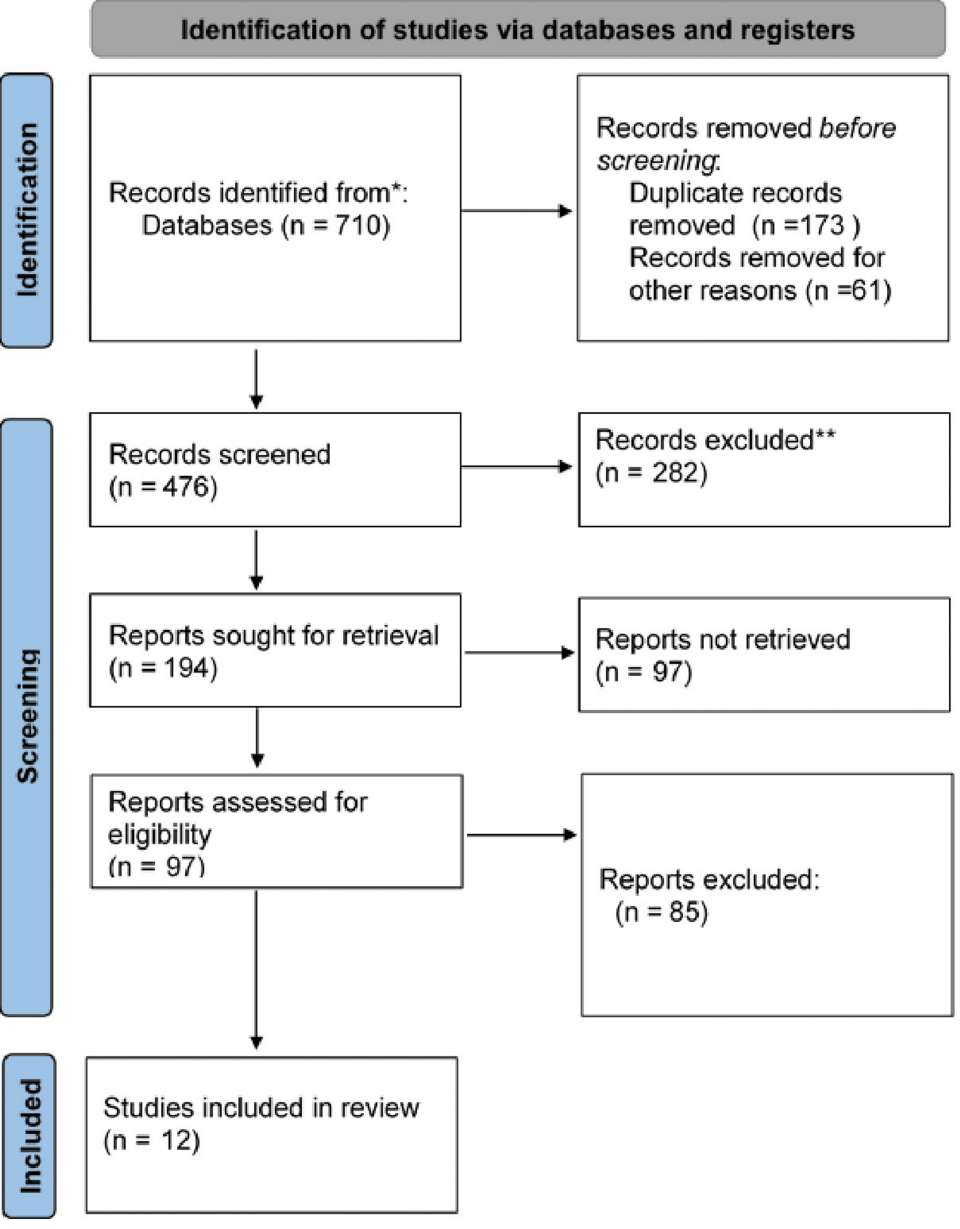
PRISMA flowchart PRISMA: Preferred Reporting Items for Systematic Reviews and Meta-Analyses

The study selection process followed the Preferred Reporting Items for Systematic Reviews and Meta-Analyses (PRISMA) guidelines. Two reviewers screened the titles and abstracts of identified studies for eligibility. Discrepancies between the reviewers were resolved by the third author through discussion. After the initial screening, full-text articles were reviewed for inclusion based on predefined eligibility criteria, which included studies that examined long-term cardiovascular or metabolic outcomes following GDM diagnosis. The pictorial presentation of the searched studies is given in Figure 1.

A total of 710 records were identified from databases, with 173 duplicates and 61 records removed for other reasons, leaving 476 records for screening. After excluding 282 records, 194 reports were sought for retrieval, of which 97 were not retrieved; 97 reports were assessed for eligibility, 85 were excluded, and 12 studies were included in the review.

Data Extraction

Data from the selected studies were extracted using a standardized data extraction form. The following information was collected for each study: author(s), publication year, study design, sample size, participant characteristics, GDM diagnostic criteria, follow-up duration, outcomes measured, and key findings related to long-term cardiovascular and metabolic health outcomes for both mothers and their offspring.

Quality Assessment

Initially, the two authors conducted the evaluation and then the third author added his input. The methodological quality of the included studies was assessed using the Newcastle-Ottawa Scale (NOS) for cohort and observational studies. The evaluation criteria focus on study participant selection methods alongside group matching protocols and outcome measurement reliability. The Cochrane Risk of Bias tool evaluated RCTs by assessing bias-related issues across several domains including random sequence generation and allocation concealment and blinding activities. Studies earned high-quality status when their NOS scores reached or exceeded seven points and lower-rated studies received moderate to low-quality designations.

Data Synthesis

The diverse range of study approaches, measurements, and demographic groups required us to use a qualitative synthesis method. Themes were identified based on maternal age, ethnicity, and diagnostic criteria. Subgroup stratification was based on these factors, allowing for targeted risk assessments. The use of Meta-analysis proved elusive because studies implemented different GDM diagnostic methods while measuring success using various indicators. The authors presented a narrative summary of their findings that used maternal and offspring results as their main groups. Subgroup analyses were carried out to assess how various interventions combined with diagnostic criteria and follow-up periods affected treatment results.

Risk of Bias and Limitations

Publication bias assessment was conducted with funnel plots and Egger's test on the included studies. The review faces several limitations including potential selection biases together with methodological variations and the restriction of foreign language publications. Studies face challenges in comparing results because diagnostic criteria for GDM as well as follow-up durations differ between research. Regional differences in GDM prevalence and outcomes were acknowledged as limitations, potentially underrepresenting certain populations.

Results

A systematic review examines the prolonged cardiovascular and metabolic health effects of GDM which impact mothers and offspring. The research examined here shows how GDM leads to damaging health effects that impact multiple demographics by demonstrating notable metabolic and cardiovascular consequences.

The impact of maternal GDM on the metabolic and cardiovascular health of offspring was also well-documented in the reviewed studies (Table 1). Children born to mothers with GDM exhibited a significantly higher risk of developing obesity, insulin resistance, and various cardiovascular abnormalities. Notably, Di Bernardo et al. [16] reported neonatal cardiac changes included structural abnormalities (e.g., ventricular hypertrophy) and functional alterations (e.g., impaired myocardial relaxation), while Venkatesh et al. [18] found that children aged 10-14 years had a higher prevalence of cardiovascular health issues, including elevated blood pressure and dyslipidemia, compared to their peers.

**Table 1:** Offspring health outcomes in relation to maternal GDM GDM: gestational diabetes mellitus

	Author(s)	Study title	Findings	Type of study	Sample size
1	Rhee et al. (2014) [1]	Metabolic health and diabetes development	Higher risk of diabetes in metabolically unhealthy individuals	Cohort study	6,748
2	Lindsay et al. (2015) [4]	Probiotics and metabolic health in GDM	No significant impact on glycemic control, but reduced LDL cholesterol	RCT	149
3	Perrine et al. (2016) [6]	Lactation and maternal cardio-metabolic health	Lactation reduces risk of diabetes and hypertension	Cohort study	Not specified
4	Di Bernardo et al. (2017) [8]	MySweetHeart cohort study	GDM linked to adverse cardiovascular health in offspring	Cohort study	200 (100 GDM, 100 non-GDM)
5	Lorenzo-Almorós et al. (2019) [10]	Predictive biomarkers for GDM	Identified biomarkers for early detection of GDM	Observational study	Not specified
6	Christensen et al. (2022) [12]	Metabolic morbidity post-GDM	GDM leads to increased metabolic disorders post-pregnancy	Register-based cohort	700,648
7	Quansah et al. (2023) [15]	CRP levels and postpartum metabolic outcomes	Elevated CRP predicts future metabolic issues post-GDM	Cohort study	211
8	Di Bernardo et al. (2023) [16]	Neonatal cardiovascular health	GDM affects newborn heart function and structure	Cohort study	123 (GDM), 141 (non-GDM)
9	Venkatesh et al. (2024) [18]	Offspring cardiovascular health in adolescence	GDM linked to worse cardiovascular health in offspring at age 10-14	Prospective cohort	3,317
10	Saravanan et al. (2020) [19]	Opportunities for improving maternal and child health	GDM management needs to consider long-term metabolic risks	Review study	Not specified
11	McKenzie-Sampson et al. (2018) [20]	Long-term cardiovascular risks	GDM increases long-term cardiovascular disease risk	Retrospective cohort	1,070,667
12	Perak et al. (2021) [21]	Cardiovascular health and pregnancy outcomes	Poor cardiovascular health during pregnancy linked to adverse outcomes	Prospective cohort	2,304

Risk of Bias Assessment

The assessment of included study quality utilized the NOS to evaluate cohort and observational research based on selection criteria and outcome measurement procedures and matched patient groups. Cohort studies received strong scores under the NOS, demonstrating sound methodological quality across most observations (>7 points). Some research studies faced potential bias issues because researchers used different methods for diagnosing GDM or did not follow subjects for similar durations. Moderate-risk studies were those with potential selection bias or unclear diagnostic criteria (e.g., one-step vs. two-step GDM testing). These inconsistencies affected comparability. The data about potential sources of bias appear in Table 2.

**Table 2:** Newcastle-Ottawa Scale (NOS) item scores and total scores

Reference (APA)	Study type	Risk of bias	Selection (0-4)	Comparability (0-2)	Outcome (0-3)	Total NOS score (0-9)
Rhee et al. (2014) [1]	Cohort	Low	4	2	3	9
Lindsay et al. (2015) [4]	RCT	Low	N/A	N/A	N/A	N/A
Perrine et al. (2016) [6]	Observational	Moderate	3	1	2	6
Di Bernardo et al. (2017) [8]	Cohort	Low	4	2	3	9
Lorenzo-Almorós et al. (2019) [10]	Observational	Low	4	2	3	9
Christensen et al. (2022) [12]	Register-based cohort	Low	4	2	3	9
Quansah et al. (2023) [15]	Cohort	Low	4	2	3	9
Di Bernardo et al. (2023) [16]	Cohort	Low	4	2	3	9
Venkatesh et al. (2024) [18]	Cohort	low	3	2	3	8
Saravanan et al. (2020) [19]	Review	N/A	N/A	N/A	N/A	N/A
McKenzie-Sampson et al. (2018) [20]	Retrospective cohort	Moderate	3	1	2	6
Perak et al. (2021) [21]	Cohort	Low	4	2	3	9

​​*Limitations*

This evaluation encountered multiple limitations stemming from the diverse study configurations and diagnostic definitions for GDM along with varied follow-ups which diminished the ability to match findings precisely. Selectivity bias was evident, as studies with null results were less likely to be published. Examining the available studies revealed that selectivity biased certain findings to remain unpublished. More research must be conducted to solve these problems.

Discussion

The systematic review results demonstrate conclusive evidence that GDM creates cardiovascular and metabolic healthcare risks for mothers and their children during and after pregnancy. Research from numerous studies establishes that GDM leads to enduring health consequences for mothers by escalating their probability of type 2 diabetes mellitus (T2DM) development and increasing their susceptibility to metabolic syndrome and hypertension. Women who have experienced GDM show type 2 diabetes risks that can be 2.5 to seven times higher than non-GDM pregnant women following childbirth according to multiple cohort studies. Multiple scientific works validate GDM as a diabetes-initiating condition in women thus creating a basis for implementing preventive control interventions from the early stages.

Hyperglycemia during pregnancy induces fetal programming, increasing long-term cardiovascular risks. Screening recommendations include annual glucose testing for post-GDM women. Heterogeneity in findings is attributed to differences in diagnostic criteria and population characteristics. Future research should standardize GDM diagnosis and investigate genetic/epigenetic mechanisms.

The established connection between GDM and obesity and hypertension among mothers confirms the importance of extensive follow-up care after a GDM diagnosis. Di Bernardo et al. [8] and Saravanan et al. [19] present evidence emphasizing that heart disease reduction for this targeted group requires pharmaceutical treatment along with ongoing medical surveillance and lifestyle modifications.

Maternal GDM diagnosis during pregnancy causes negative health impacts on their offspring. During pregnancy when blood sugar levels are uncontrolled, newborns face an increased risk of developing obesity and insulin resistance along with potential future cardiovascular diseases. The study by Di Bernardo et al. [16] demonstrated how GDM causes heart functionality alterations among newborns who experienced maternal GDM thus showing that GDM conditions extend past delivery. Scientific evidence published by Venkatesh et al. [18] demonstrates that children aged 10 to 14 who had mothers with gestational diabetes exhibit abnormal blood pressure readings and dyslipidemic patterns when compared to children without gestational diabetes exposure. Future health complications demand preventive measures because GDM risks impact both maternal well-being and infant outcomes according to research findings.

This research review suffers from many weaknesses that reduce the validity of its findings. Many dissimilar research outcomes stem from distinct follow-up durations and different research approaches and medical diagnostic methods used across various studies. Results from the asymmetrical funnel test show evidence that research reporting might disregard studies with no GDM findings or negative results, which suggests negligence in accurately measuring GDM impacts on maternal health outcomes. This extensive review highlights the need to continue maternal and newborn surveillance under intervention programs to manage cardiovascular and metabolic complications before they become chronic health issues in high-risk patient populations.

## Conclusions

This systematic review shows that GDM generates multiple cardiovascular and metabolic complications that affect both mothers and their newborns. Both type 2 diabetes and hypertension plus metabolic syndrome occur more frequently in maternal GDM patients leading their offspring to encounter elevated risks of heart disease abnormalities, weight management problems, and insulin resistance concerns. Correct and timely GDM diagnosis needs ongoing care management with extended support plans to help patients who have GDM. Standardized diagnostic protocols and intervention procedures function as crucial elements for reducing potential risks to patients. Research limitations and inconsistent follow-up periods do not undermine solid study findings that prove GDM's lasting effects on mothers along with their children's health. The investigation of predictive biomarkers and evaluations of effective interventions and lifestyle-risk reduction approaches need to be studied together as a base for clinical care and public health policy development. Risks of T2DM, hypertension, and metabolic syndrome post-GDM are 2.5 to seven times higher. Offspring exhibit increased rates of obesity, hypertension, and dyslipidemia. Public health strategies should include resource allocation for long-term monitoring and preventive programs. Future studies should focus on generational impacts and RCTs for intervention efficacy.
